# Congruence of morphological and molecular phylogenies of the rove beetle subfamily Staphylininae (Coleoptera: Staphylinidae)

**DOI:** 10.1038/s41598-019-51408-1

**Published:** 2019-10-22

**Authors:** Chen-Yang Cai, Yong-Li Wang, Lü Liang, Zi-Wei Yin, Margaret K. Thayer, Alfred F. Newton, Yu-Lingzi Zhou

**Affiliations:** 1 State Key Laboratory of Palaeobiology and Stratigraphy, Nanjing Institute of Geology and Palaeontology and Center for Excellence in Life and Paleoenvironment, Chinese Academy of Sciences, Nanjing, 210008 China; 2 School of Earth Sciences, University of Bristol, Life Sciences Building, Tyndall Avenue, Bristol, BS8 1TQ UK; 3 Biofuels Institute, School of the Environment and Safety Engineering, Jiangsu University, Zhenjiang, 212013 China; 4 College of Life Sciences, Hehei Normal University, Shijiazhuang, 050024 China; 5 Lab of Environmental Entomology, College of Life Sciences, Shanghai Normal University, Shanghai, 200234 China; 6 Integrative Research Center, Field Museum of Natural History, Chicago, IL 60605 USA; 7 Key Laboratory of Zoological Systematics and Evolution, Institute of Zoology, Chinese Academy of Sciences, Beijing, China; 8 Australian National Insect Collection, CSIRO, Canberra, Australia

**Keywords:** Bayesian inference, Entomology

## Abstract

Staphylininae is the third largest subfamily of the enormous family Staphylinidae. Monophyly of Staphylininae and its sister relationship to the subfamily Paederinae have been broadly accepted according to both conventional morphology- and molecular-based phylogenies until the last three years. Recent molecular phylogenies rejected monophyly of Staphylininae and regarded Paederinae as a clade within it. This paper re-evaluates the recent molecular work, aiming to clarify the relationship between Staphylininae and Paederinae and resolve intertribal relationships within Staphylininae. Based on a new six-gene data set (5707 bp) for 92 taxa including Oxyporinae (outgroup), representatives of Paederinae, and members of all extant tribes of Staphylininae from published DNA data in GenBank, we generated a well-resolved phylogeny of Staphylininae with all deep nodes (intertribal relationships) strongly supported, and reassert the hypothesis that Staphylininae is monophyletic and indeed the sister group to Paederinae using both Bayesian and maximum likelihood inference. Additionally, our study is a case-study to show that both outgroup selection and completeness of nucleotide data can influence the outcome of a molecular phylogeny. With an increasing number of staphylinid fossils being discovered, the robust phylogeny of Staphylininae inferred by our research will provide a good framework for understanding the early evolution of this group.

## Introduction

With 7972 described species grouped in 350 genera, Staphylininae Latreille, 1802 are the third most speciose rove beetle subfamily, after Aleocharinae Fleming, 1821 and Pselaphinae Latreille, 1802^1^. Staphylinine rove beetles are relatively large, fairly robust blunt-headed and short elytra, sometimes colourful, and frequently collected by naturalists. Staphylininae currently encompass seven extant tribes, viz., Arrowinini, Diochini, Maorothiini, Othiini, Platyprosopini, Staphylinini, and Xantholinini. Solodovnikov *et al*.^2^ described an extinct tribe Thayeralinini, representing a stem lineage of Staphylininae and displaying morphological character combinations transitional between extant tribes of Staphylininae and even between Staphylininae and Paederinae. Staphylininae and the closely related Paederinae have been placed in the informal Staphylinine group of subfamilies^1,3^.

**Figure 1:**
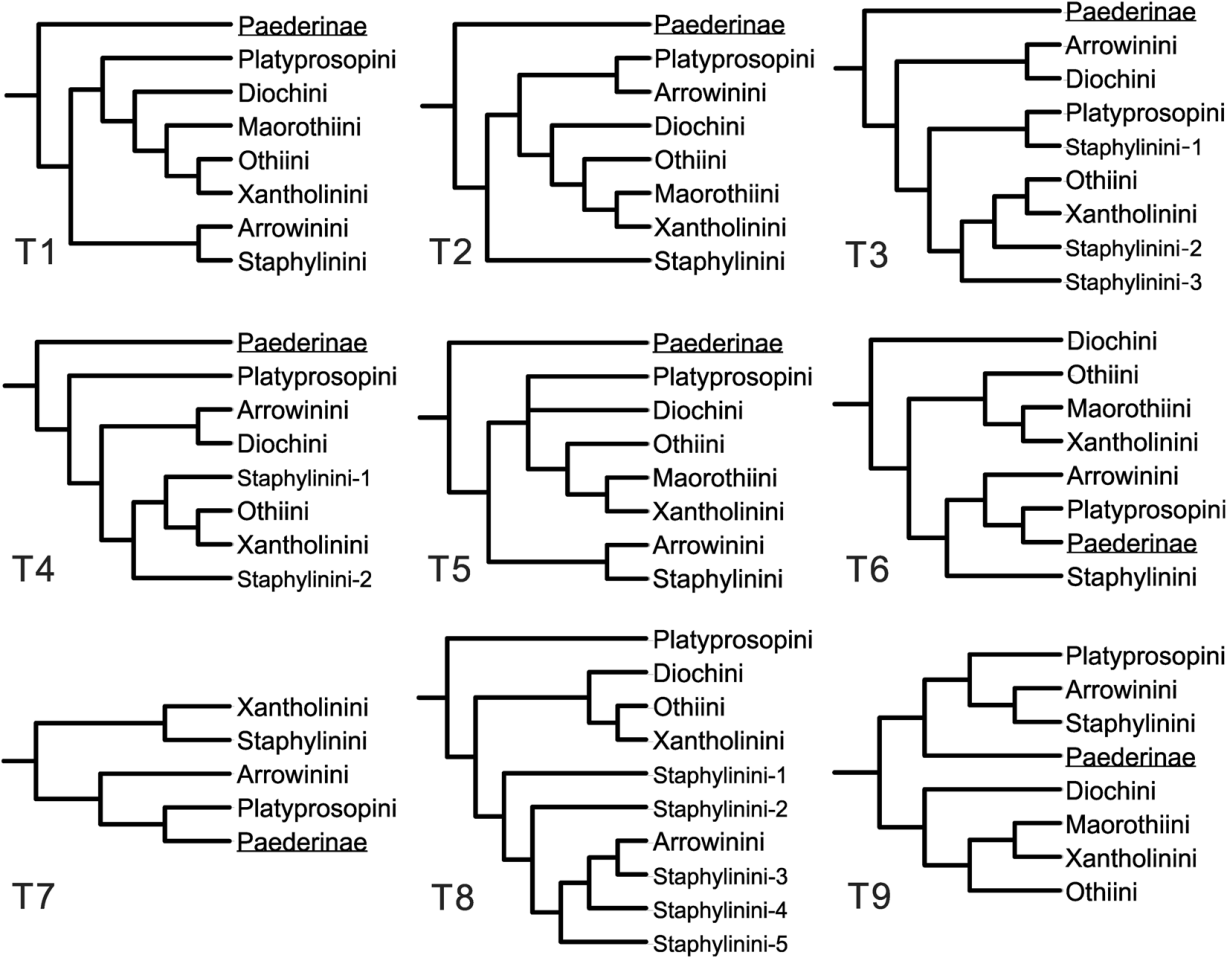
Nine proposed topologies between Paederinae and the extant tribes of Staphylininae. Topologies are derived from: T1 (phylogeny based on adult and larval characters, Solodovnikov & Newton^7^); T2 (phylogeny based on larval characters only, Solodovnikov & Newton^7^); T3 (first molecular phylogeny of Staphylininae (Bayesian inference) based on four genes; Chatzimanolis *et al*.^12^); T4 (first molecular phylogeny of Staphylininae (combined parsimony analysis); Chatzimanolis *et al*.^12^); T5 (molecular phylogeny based on two genes; McKenna *et al*.^4^); T6 (molecular phylogeny of Staphylininae based on six genes; Brunke *et al*.^14^); T7 (molecular phylogeny of Paederinae and some Staphylininae based on five genes; Schomann & Solodovnikov^17^); T8 (molecular phylogeny of Staphylininae based on four genes; Zhang & Zhou^16^); and T9 (parsimonious tree of extant and extinct taxa; Kypke *et al*.^11^). Note that the subfamily Staphylininae are polyphyletic in T6, T7 and T9.

A close affinity between Paederinae Fleming, 1821 and Staphylininae is supported by some apomorphies of both adults (metacoxae projecting medially, with posterior margin of metaventrite strongly sinuate; and adult protrochantin flat, blade-like, and protruding) and larvae (mandibles without preapical teeth, and abdominal terga and sterna longitudinally divided medially by membranous area)^1^, and by a two-gene-based phylogeny using Bayesian inference^4^. Both subfamilies have long been regarded as distinct and monophyletic groups^1,3,5^. Although there is a lack of a wide phylogenetic analysis of the hyperdiverse Paederinae, the phylogenetic relationships among tribes and key genera of Staphylininae have been extensively explored based on both morphological characters^2,6–11^ and molecular data^4,12–16^. However, it is quite unexpected that the emerging evidence showed no consensus between the morphology- and molecular-based phylogenetic analyses regarding the relationship between Staphylininae and Paederinae nor the interrelationships among extant tribes of Staphylininae (summarized in Kypke *et al*.^11^; Fig. 1).

As proposed in Kypke *et al*.^11^ the hypothesis of a paraphyletic Staphylininae was partly supported in a morphology-based phylogeny by Solodovnikov *et al*.^2^, in which multiple early Cretaceous staphylinine fossils were analyzed together with extant taxa. Surprisingly, recent molecular phylogenies of Staphylininae based on six genes strongly (Bayesian inference) to moderately (maximum likelihood) rejected both monophyly of Staphylininae, because of a sister group relationship between Platyprosopini (Staphylininae) and Paederinae, and the basal-most position of the ‘Xantholinine-lineage’ of Staphylininae, although that study was principally focused on the tribe Staphylinini^14^. Moreover, the monophyly of Staphylininae was again rejected in another molecular of DNA-based study^17^, although the goal of that study was to assess the position of the paederine genus *Hyperomma* Fauvel. Recently, Kypke *et al*.^11^ described an extinct genus *Vetatrecus* with two species from mid-Cretaceous Burmese amber (*ca*. 99 Ma). Integrated phylogenetic analyses of extant representatives of Staphylininae and Paederinae, as well as the transitional *Vetatrecus*, demonstrated the first morphology-based evidence for the paraphyly of Staphylininae with respect to Paederinae. The emerging paraphyletic Staphylininae hypothesis based on recent molecular and fossil-integrated morphology-based phylogenies conflicts strongly with the conventional morphological studies of adults and larvae^3,7^ and a two-gene based molecular phylogeny^4^.

As with the relationship between Staphylininae and Paederinae, the intertribal relationships of Staphylininae remain elusive, with little work focused primarily on this topic. The first comprehensive phylogenies testing the intertribal relationships of Staphylininae were based on morphological characters, although they were designed to place the New Zealand genus *Maorothius* Assing (tribe Maorothiini)^6^, or the South Africa genus *Arrowinus* Bernhauer (tribe Arrowinini)^7^. It is noteworthy that the phylogenetic analyses based on different datasets (adult and larval characters, and larval characters only) in the latter study yielded obviously different topologies, despite the fact that monophyletic Staphylininae were recovered in both analyses. Solodovnikov *et al*.^2^ integrated diverse Cretaceous fossils into the adult morphology-based character matrix from Solodovnikov & Newton^7^, and therefore a very similar topology was produced as expected, and a new extinct tribe Thayeralinini was established. The first molecular phylogeny of the subfamily based on four gene fragments (*COI*, *wingless*, *topoisomerase I* and *28S*) was focused on the most diverse tribe Staphylinini, and the tribe Maorothiini was not included^12^. In sharp contrast to the previously published morphology-based phylogeny^7^, the molecular phylogenies using parsimony and Bayesian analyses placed the tribes Othiini and Xantholinini nested within Staphylinini, and Platyprosopini was also recovered as a group within Staphylinini under Bayesian inference. McKenna *et al*.^4^ reconstructed the phylogeny of Staphylinidae and even the series Staphyliniformia and Scarabaeiformia using a new set of DNA sequences, but the relationships above the tribal level were poorly resolved because of limited gene sampling (only two: nuclear *28S* rDNA and nuclear protein-coding gene *CAD*). In particular, the positions of basal staphylinine tribes remained unresolved (Diochini and Platyprosopini) or weakly supported (Arrowinini) under Bayesian inference, and they were completely unresolved under maximum likelihood analysis. Unlike the results in Chatzimanolis *et al*.^12^, Staphylinini was recovered as a monophyletic group, and Diochini, Othiini and Xantholinini were recovered as isolated lineages sister to Arrowinini + Staphylinini^4^. Brunke *et al*.^14^ provided a more comprehensive molecular phylogeny of Staphylininae based on six genes, which were partially derived from the data first published by Chatzimanolis *et al*.^12^. Their results rejected the monophyly of Staphylininae and recovered Diochini as sister to other staphylinine tribes and the subfamily Paederinae. Such an unexpected result was further confirmed by Schomann & Solodovnikov^17^, although Staphylininae were represented there by only four extant tribes. In contrast to Brunke *et al*.^14^, another molecular phylogeny based on four genes by Zhang & Zhou^16^ rejected the monophyly of Staphylinini, with Arrowinini nested within it, but the ‘Xantholinini-lineage’ (Diochini, Othiini and Xantholinini) was supported. Recently, the discovery of the Cretaceous genus *Vetatrecus* (Othiini) and an integrated morphology-based phylogeny combing both extant and extinct taxa further complicated the intertribal relationships of the subfamily Staphylininae^11^.

In this study, we present a novel molecular phylogeny of Staphylininae by mining published DNA data from GenBank. We developed and analyzed a six-gene data set incorporating 92 taxa, including Oxyporinae (outgroup), representatives of Paederinae, and members of all extant tribes of Staphylininae. Additionally, in order to evaluate the congruence between the molecular and morphology-based phylogenies, we re-analyzed the morphology-based (both adult and larval characters) data set from Solodovnikov & Newton^7^ and the integrated data set from Kypke *et al*.^11^ using both maximum parsimony and Bayesian inference methods.

## Materials and Methods

### DNA-based phylogenetic analyses

#### Taxon selection

The present molecular-based study is designed to test the monophyly of Staphylininae and resolve the intertribal relationships of the subfamily Staphylininae. Therefore, we conducted a broad sampling of the taxa of staphylinine tribes, including representatives of as many genera of all extant tribes as were currently available in GenBank. All extant tribes of Staphylininae were assembled: the monogeneric tribes Arrowinini (1 genus, 2 species), Maorothiini (1 genus, 1 species) and Platyprosopini (1 genus), Diochini (represented by two species of *Diochus* combined), Othiini (represented by 2 genera and 3 species), Xantholinini (represented by 4 genera and 4 species), and Staphylinini (represented by 43 genera and 72 species, covering nearly all extant subtribes). As in Brunke *et al*. (2016), six gene fragments were used for phylogenetic analyses based on their contribution to resolving both deep and shallow divergences within Staphylinidae^13^: the nuclear protein-coding genes *arginine kinase* (*ArgK*), *carbamoyl-phosphate synthetase* (*CAD*), *topoisomerase I* (*TP*), *wingless* (*Wg*); mitochondrial protein-coding *cytochrome oxidase I* (*COI*); and nuclear ribosomal *28S*. The genes of one of the studied taxa (Diochini, as *Diochus* spp.) were assembled from two species: *28S* from *Diochus schaumi*^4^ and the other five from a Neotropical species of *Diochus*^14^. Similarly, the *28S* gene of *Platyprosopus* was sequenced by McKenna *et al*.^4^, whereas the remaining five genes were derived from Chatzimanolis *et al*.^12^ and Chatzimanolis^13^, but both were based on co-collected South African specimens presumed to be the same species. The gene and taxon sampling of the diverse Staphylinini followed Brunke *et al*.^14^, as those species represent the majority lineages of the tribe. We did not include more taxa belonging to Staphylinini recently published by Chani-Posse *et al*.^15^, because that study was aimed at the subtribe Philonthina of the derived ‘Staphylinini propria’, which is not in the scope of our study. The genes of six paederines and the outgroup (Oxyporinae) were from Brunke *et al*.^14^ and Schomann & Solodovnikov^17^. GenBank accession numbers for the analyzed sequences are given in Table S1.

#### Sequence assembly, management and alignment

All gene sequences were retrieved by using the online Batch Entrez from GenBank. GenBank accession numbers of all DNA sequences are given in Table S1. Sequences were aligned using the MAFFT v.7.2 software^18,19^. The unclear ribosomal *28S* gene was aligned using the Q-INS-I algorithm of MAFFT. Protein-coding genes were unambiguously aligned based on their codon-based structure. The GBlocks masking method^20^ for alignment-variable regions was not applied, unlike the method used by Brunke *et al*.^14^. Six gene alignments were concatenated using SequenceMatrix^21^.

#### Data partitions and model selection

The nucleotide sequence alignments for the six genes were concatenated, producing a super-matrix containing 5707 nucleotide positions for 92 species. To select the best data-partitioning scheme and best-fitting models of nucleotide substitution, we used the greedy algorithm in PartitionFinder2^22^. The supermatrix was analyzed with 16 partitions (*28S* and five protein-coding genes partitioned by codon position). The best-fit substitution model for each partition was selected using the Bayesian Information Criterion. The partitioning scheme and corresponding models selected were: (1) *28S*: SYM + G; (2) position 1 of *ArgK*: SYM + I + G; (3) position 2 of *ArgK*: TVMEF + I; (4) position 3 of *ArgK*: GTR + I + G; (5) position 1 of *CAD*, *TP* and *Wg*: SYM + I + G; (6) position 2 of *CAD*, *TP* and *Wg*: GTR + I + G; (7) position 3 of *CAD*: GTR + I + G; (8) position 1 of *CO1*: GTR + I + G; (9) position 2 of *CO1*: GTR + I + G; (10) position 3 of *CO1*: HKY + I + G; (11) position 3 of *TP*: TVMEF + I + G; and (12) position 3 of *Wg*: TRN + I + G.

#### Phylogenetic analyses

Phylogenetic analyses were performed using Bayesian inference (BI) and maximum likelihood (ML) methods to examine topological congruence and potential conflict. The Maximum parsimony (MP) approach was not used, because it has been well known that model-based approaches based on ML inferences (including BI) outperform parsimony in molecular phylogenetic analyses^23,24^. BI was conducted in MrBayes v.3.2.3^25^. Each run consisted of one cold and three heated chains, ran for 25 million generations and was repeated twice. ML analysis was performed using IQ-TREE v.1.6.1^26^. Node support was assessed by performing 1000 bootstrap pseudoreplicates. Clades with Bayesian posterior probability (BPP) > 0.95 or ML bootstrap (BS) values > 80 were considered strongly supported; clades with PP = 0.90–0.94 or BS = 70–80 were considered moderately supported; clades with PP = 0.85–0.89 or BS = 50–69 were considered to be weakly supported; and clades with PP < 0.85 or BS < 50 were regarded as unsupported.

### Morphology-based phylogenetic analyses

#### Data matrix assembly

We reproduced two data matrices based on Solodovnikov & Newton^7^: one with both adult and larval characters (18 taxa, 100 characters), and the other with larval features only (18 taxa, 33 characters). In addition, we regenerated the data from Kypke *et al*.^11^, which includes 36 taxa and 76 morphological characters).

#### Phylogenetic analyses

Bayesian analyses were carried out using MrBayes v.3.2.3 and the discrete Mkv + G model^27^. For each data set, two MCMC runs of four chains were run for three million generations. The consensus tree was estimated after a burn-in of 25% of the sampled trees. Convergence was confirmed with Tracer version 1.6.0^28^. Maximum parsimony (MP) analysis was conducted using TNT 1.5^29^ using the New Technology search. Two search strategies were selected: equal weights and implied weights. For the implied weights analyses, we adopted a concavity (*k*) value of 12 as suggested by Goloboff *et al*.^30^ Branch support values were estimated using 10,000 bootstrap replicates. Levels of support were assessed as above for the ML analyses.

## Results

### Molecular phylogenetic trees and clade support

**Figure 2:**
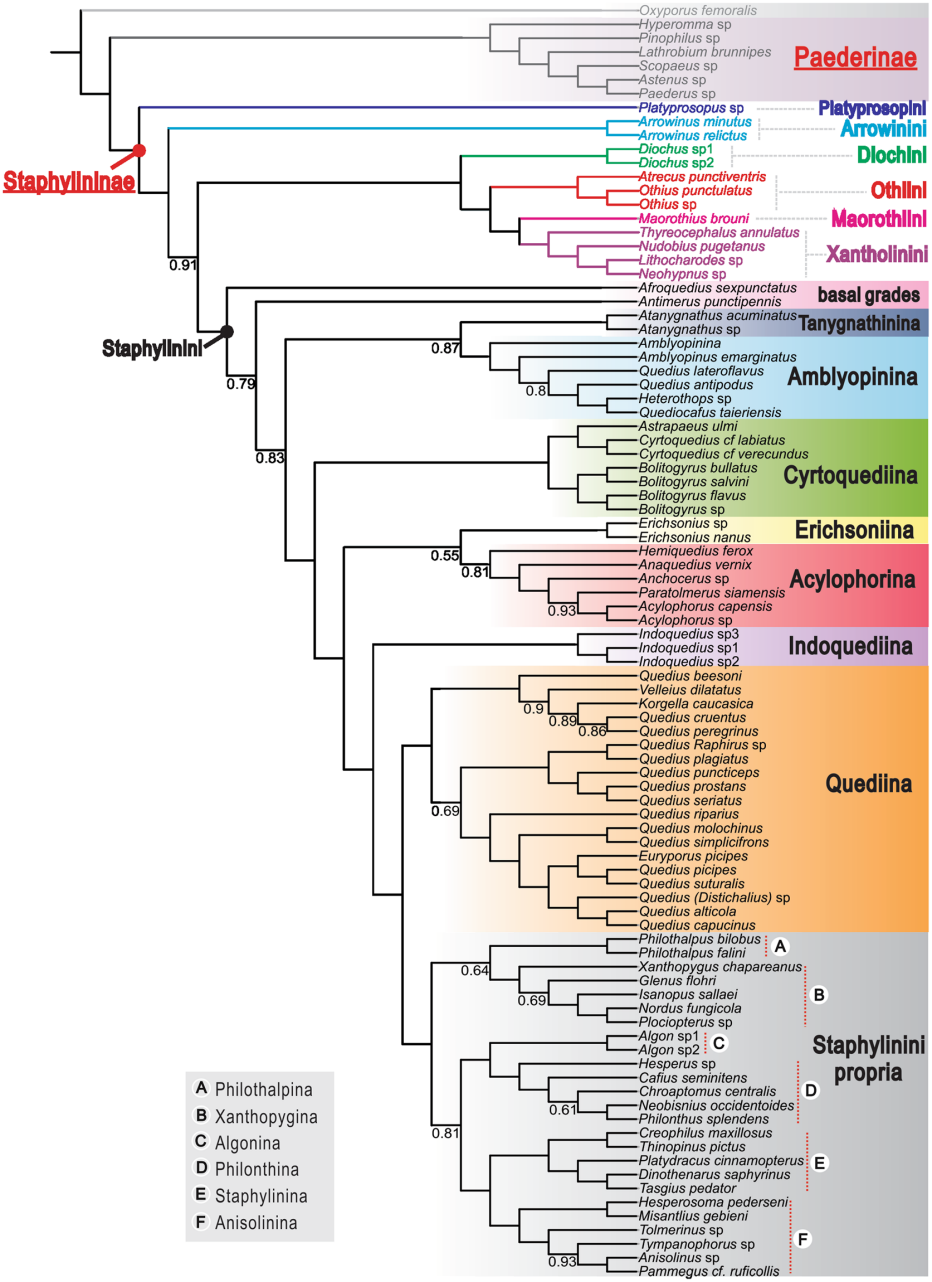
Fifty per cent majority rules consensus tree from a partitioned Bayesian analysis of six genes, with posterior probabilities smaller than 0.95 reported below the node. Unlabelled nodes are strongly supported (BPP > 0.95).

We estimated the phylogeny from the concatenated nucleotide data set using both BI and ML approaches. The phylogenetic hypothesis for the subfamily Staphylininae in Fig. 2 shows relationships generated from Bayesian analysis. The ML tree (Fig. S1) with the same partitions and models as in the BI analysis resulted from a likelihood analysis of the same dataset. The clade support estimated under BI (BPP) and ML (BS) are summarized on the resulting trees. The BI analysis generated a well-resolved phylogeny of Staphylininae, with all deeper nodes (intertribal relationships) being strongly supported. The topology obtained from ML analysis is nearly identical to the BI one, with only one discrepancy within the subtribe Xanthopygina. The strengths of branch support in the BI and ML analyses are equivalent or nearly so.

### Higher-level relationships and interrelationships of the tribes of Staphylininae

The subfamilies Staphylininae and Paederinae were strongly supported as monophyletic groups in both BI and ML trees. All nodes within Paederinae were resolved with strong support (BPP > 0.95; BS > 95). The relationships within Paederinae, a diverse group of Staphylinidae, are outside the scope of the present study, as we focused on the relationships within Staphylininae. All staphylinine tribes were recovered as monophyletic with strong support. The intertribal relationships of Staphylininae recovered in the BI and ML trees were identical –Platyprosopini (Arrowinini ((Diochini (Othiini (Maorothiini, Xantholinini))) Staphylinini)) (Figs 2 and S1). All tribes received maximal BPP support and strong to maximal BS support. The support for (Diochini (Othiini (Maorothiini, Xantholinini))) + Staphylinini was slightly weaker (BPP = 0.91; BS = 84) compared to deeper node support, but the clade was still moderately (BI) to strongly (ML) supported.

### Relationships within staphylinini

Staphylinini were strongly supported as monophyletic in BI and ML analyses (BPP = 1, BS = 99). The South African endemic *Afroquedius* Solodovnikov was recovered as the first divergence within Staphylinini, but with weak support for the remainder of the tribe only in the ML tree (BS = 64). The Australian endemic *Antimerus* Fauvel was sister to the remaining majority of Staphylinini that are classified in subtribes, but the latter group was weakly supported only in the ML tree (BS = 62). The subtribes Tanygnathinina (*Atanygnathus* Jakobson) and Amblyopinina were each recovered as monophyletic with maximal support in both analyses. A sister relationship between Tanygnathinina and Amblyopinina was moderately supported in the ML tree (BS = 74), but weakly supported in the BI tree (BPP = 0.87). The subtribe Cyrtoquediina (*Astrapaeus* Gravenhorst, *Bolitogyrus* Chevrolat and *Cyrtoquedius* Blackwelder) was maximally supported as monophyletic (BPP = 1, BS = 100) and all nodes within Cyrtoquediina also received maximal support in both trees. *Bolitogyrus*, represented by Neotropical and Oriental lineages, was maximally supported as monophyletic. *Erichsonius* (Erichsoniina) was recovered as monophyletic in both analyses, and sister to the subtribe Acylophorina, but with weak support in only the ML tree (BS = 62). Acylophorina was recovered as monophyletic with strong support (BS = 81) in only the ML analysis. The monophyly of *Indoquedius* Blackwelder was maximally supported (BPP = 1, BS = 100), and the subtribe was recovered as the sister group to Quediina + Staphylinini propria with strong support in both analyses (BPP = 0.99, BS = 94). Quediina was maximally supported as monophyletic (BPP = 1, BS = 100), and strongly (BPP = 1, BS = 91) as sister group to a monophyletic Staphylinini propria. As found in Brunke *et al*. (2016), three strongly supported clades were recovered within Quediina by both methods. Within Staphylinini propria, all sampled subtribes (Algonina, Anisolinina, Philothalpina, Philonthina, Staphylinina and Xanthopygina) were recovered as monophyletic, with maximal support in the BI and ML trees except for Anisolinina in the ML result (BS = 98). A sister relationship between Algonina and Philonthina was strongly supported (BPP = 0.99, BS = 93). Anisolinina was strongly supported as sister to Staphylinina (BPP = 1, BS = 94). The clade Algonina + Philonthina was recovered as the sister group to Anisolinina + Staphylinina only in the ML tree (BS = 81).

### Morphology-based phylogeny using dataset from Solodovnikov & Newton (2005)

**Figure 3:**
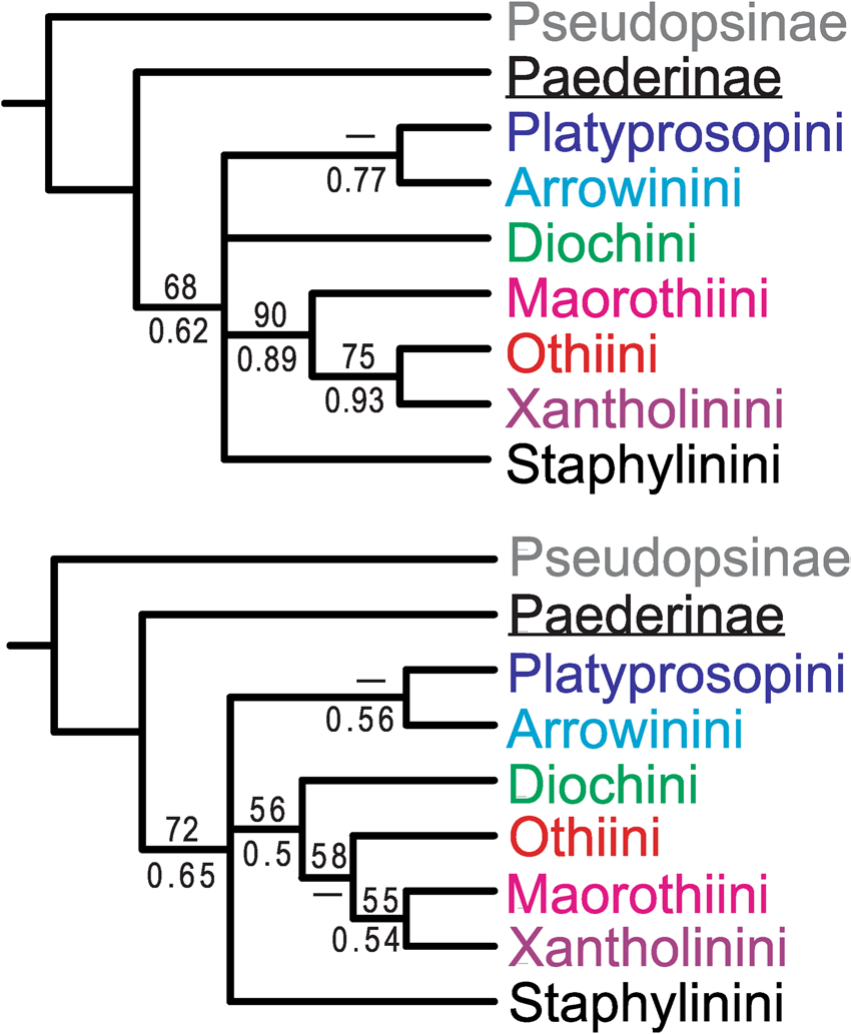
Summarized topologies of the morphology-based phylogenies using the datasets from Solodovnikov & Newton (2005). Upper: topology based on adult and larval characters; lower: topology based on larval characters only. BPP (Bayesian inference) and BS (implied weighting parsimony) are shown below and above each node, respectively.

Based on the dataset with adult and larval characters, both Paederinae and Staphylininae were recovered as monophyletic groups, although Staphylininae was not strongly supported in either tree (BS = 68 [implied weighting]; BPP = 0.62) (Fig. 3, upper). A sister-group relationship between Platyprosopini and Arrowinini was found in only the BI tree but unsupported (BPP = 0.77). The clade (Maorothiini, (Othiini, Xantholinini)) was recovered with strong to weak support (BS = 90; BPP = 0.89). The intertribal relationships within Staphylininae were largely unresolved.

The trees resulting from the larval characters alone (Fig. 3, lower) revealed monophyletic Paederinae and Staphylininae, but monophyly of the latter subfamily was only moderately supported or unsupported (BS = 72; BPP = 0.65). The intertribal relationships within the Xantholinini-lineage were differently resolved, but more weakly supported. Diochini was weakly supported as sister to the clade (Othiini (Maorothiini, Xantholinini)) in only the MP tree (BS = 56) under implied weighting (*k = *12). Maorothiini was recovered as sister group to Xantholinini but with at most weak support (BS = 55; BPP = 0.54). Monophyly of tribe Staphylinini was not recovered in the equally weighted MP analysis.

### Morphology-based phylogeny using dataset from Kypke *et al*. (2018)

**Figure 4:**
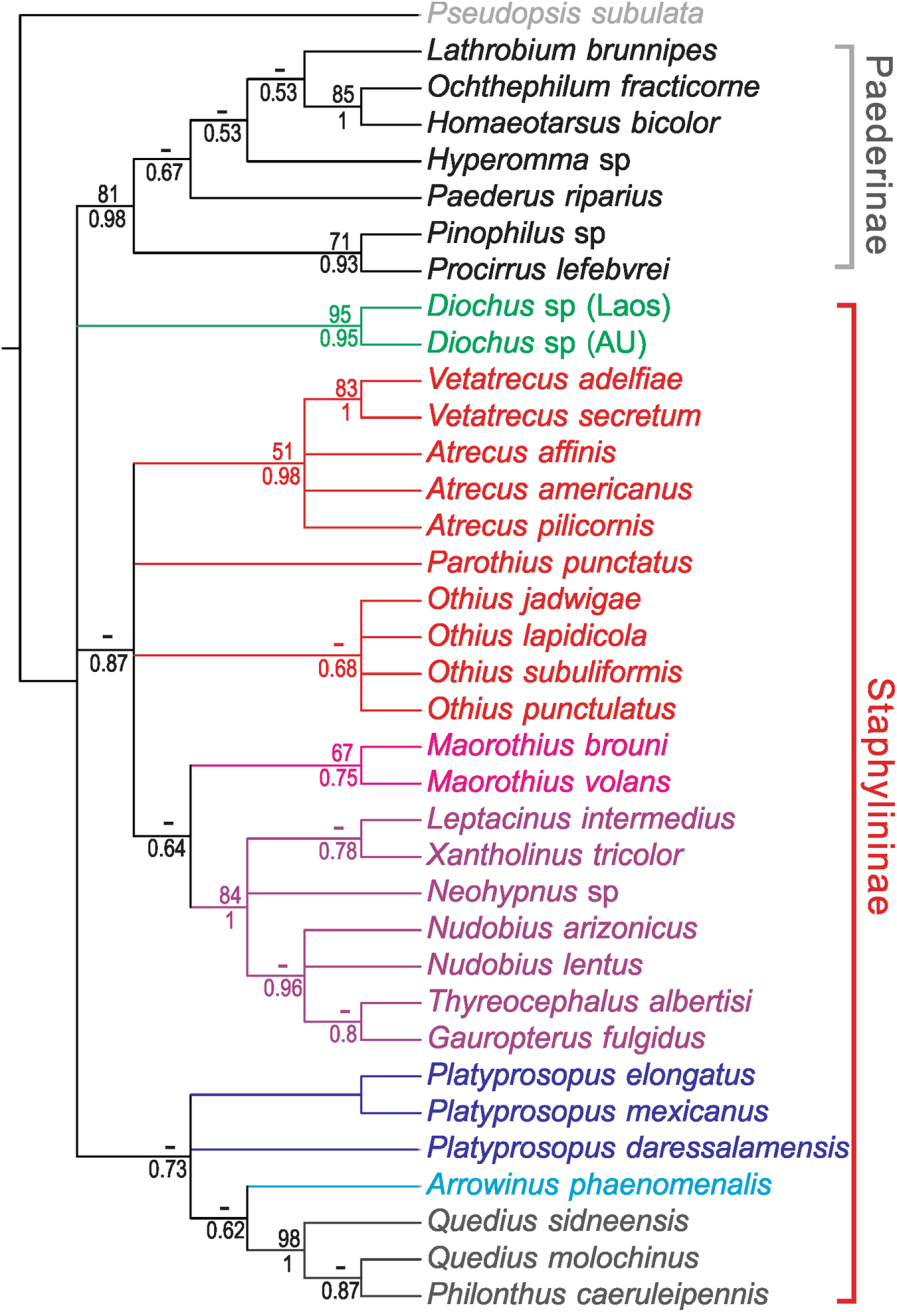
Summarized topology of the morphology-based phylogenies using the dataset from Kypke *et al*.^11^. BS under equally weighted parsimony (above) and BPP (below) are given at each node. Tribes are color-coded.

In the MP analysis under equal weights, one most parsimonious tree with 234 steps resulted (Fig. 4)^11^. Bootstrap analysis found only Paederinae strongly supported as a monophyletic group (BS = 81). The relationships among staphylinine tribes were not resolved. Most lineages of Staphylininae were recovered as a polytomy, but the tribes Diochini, Maorothiini, Staphylinini and Xantholinini were supported as monophyletic (BS = 95, 67, 98, 84). Neither Othiini nor Platyprosopini was recovered as monophyletic. A nearly identical topology was recovered in the implied weighting MP analysis (*k* = 12), but monophyly of Platyprosopini was weakly supported (BS = 67).

In the BI tree (Fig. 4), intertribal relationships within Staphylininae were slightly better resolved than in the MP analysis. As in the MP tree, monophyly of Paederinae was strongly supported (BPP = 0.98), but Staphylininae was not resolved. The tribes supported in the MP analysis (Xantholinini, Staphylinini) were also supported in the BI tree. Although a monophyletic Othiini was not supported, the clade Othiini + Maorothiini + Xantholinini was weakly supported (BPP = 0.87) and *Vetatrecus* + *Atrecus* was strongly supported (BPP = 0.98).

## Discussion

### Comparisons between datasets in Brunke *et al*.^14^ and this study

Our newly assembled dataset analyzed herein is largely consistent with the one present in Brunke *et al*.^14^, so the contradicting results (e.g., monophyly of Staphylininae, intertribal relationships of Staphylininae) are unexpected. The main differences between the data matrices of Brunke *et al*.^14^ and the present study are: 1) more gene sequences were added in our study (*28S* gene fragments for the species of *Diochus* and *Platyprosopus*; four paederine species belonging to four genera (*Lathrobium*, *Paederus*, *Pinophilus* and *Scopaeus*) to maintain the completeness of six genes of the Paederinae species); and 2) one of the two outgroups in Brunke *et al*.^14^, a species of *Pseudopsis* Newman (Pseudopsinae), was excluded from the present study.

Why was *Pseudopsis* sp. excluded from our analyses? To test the impact of inclusion of *Pseudopsis* sp. for reconstructing the phylogeny of Staphylininae, we also analyzed a 93-taxon (5707 bp) data set (test phylogeny), with two species of *Pseudopsis* in Brunke *et al*.^14^ integrated into one taxon (as *Pseudopsis* sp.) to improve the gene coverage. Using *Oxyporus femoralis* as the outgroup, Paederinae and Staphylininae formed a sister group, but *Pseudopsis* clustered with the staphylinine genus *Diochus* in the ML tree (Fig. S2). It is puzzling that the result shown in Brunke *et al*.^14^, *Oxyporus femoralis* and *Pseudopsis* forming outgroups, was not repeated in our BI or ML trees. The subfamily Pseudopsinae, morphologically well characterized by epipharynx with globosetae and abdominal tergite IX with lateral stridulatory files in the adults^1,3^ is a distinctive and isolated lineage of Staphylinidae. The unexpected relationship between *Pseudopsis* and *Diochus* is probably explained by the limited gene sampling of the former taxon. As shown in Brunke *et al*.^14^, the species of *Pseudopsis* were only represented by five genes (*ArgK*, *CAD*, *TP*, *COI* and *28S*), but the key gene *Wg*, most critical and informative for elucidating the relationships above the level of subtribe^14^, was absent in all analyses. Therefore, in our test phylogeny, the systematic position of *Pseudopsis* (as a sister group to *Diochus*) was probably inaccurate and misleading. In contrast, the species of *Oxyporus* was represented by all six gene fragments, so it can be used as a reliable outgroup, given the fact that we intended to test the monophyly of Staphylininae and the intertribal relationships. Further sampling of more genes of the genus *Pseudopsis*, especially *Wg*, will be important for better understanding the relationship between Staphylininae and Paederinae.

### Monophyly of paederinae and staphylininae

The families Paederinae and Staphylininae were each strongly supported as monophyletic, as indicated by morphological characters^1,3,7^ and one previously published molecular phylogeny^4^. The monophyly of Paederinae + Staphylininae cannot be confirmed herein, because there is a lack of reliable genes for the putatively closely related subfamily Pseudopsinae. As conventionally defined, the monophyly of Paederinae is also supported by a set of unique larval traits, such as the presence of one pair of trichobothria each on head, stipes, and pronotum; unsclerotized and setose ligula; and two (rather than three) larval instars^1^.

The monophyly of Staphylininae recovered here strongly contradicts the hypothesis recently proposed by Brunke *et al*.^14^, Schomann and Solodovnikov^17^, and Kypke *et al*.^11^, but is consistent with the long-standing view derived from morphological characters. Staphylininae, as a sister group to Paederinae, has long been regarded as monophyletic, which was supported by morphology-based phylogenetic analyses^3,6,7^. In particular, staphylinines have an obtect pupa and larvae with a large triangular eusternum on the prothorax, both characters unique within Staphylinidae^1,19^. In addition, they lack the typical brick-wall pattern of minute sclerites on the abdominal intersegmental membranes of adults (except *Arrowinus*)^1^. As such, non-monophyly of Staphylininae is ‘difficult to explain’, especially as the genes used by McKenna *et al*.^4^ were a small subset of those used by Brunke *et al*.^14^. Brunke *et al*.^14^ pointed out that such a conflict resulted from the absence of *Wg* in the dataset used by McKenna *et al*.^4^, which provided the greatest number of synapomorphic base-pair substitutions for the clades in conflict. However, our new analyses using almost the same dataset as that in Brunke *et al*.^14^ generated a topology more consistent with that in McKenna *et al*.^4^, i.e., Paederinae and Staphylininae were both recovered as monophyletic groups.

Another molecular-based phylogeny of the Paederinae that included several staphylinine taxa also recovered a non-monophyletic Staphylininae^17^. In that paper, the relationships among the staphylinine tribes were largely unresolved. As discussed above, this probably resulted from selection of an outgroup (*Pseudopsis* sp.), that lacked data for the most informative gene (*Wg)* for reconstructing the phylogeny above the level of subtribe and limited taxon sampling within Staphylininae. Without a reliable outgroup for rooting the phylogenetic tree, the basal relationships in the tree topology shown in Schomann & Solodovnikov^17^ were probably misleading.

Recently, Kypke *et al*.^11^ presented the first morphology-based evidence for paraphyly of Staphylininae with respect to Paederinae, based on an integrated morphology-based phylogeny of extant representatives of Paederinae and Staphylininae, as well as a Cretaceous othiine genus *Vetatrecus*. We re-analyzed the same data set from Kypke *et al*.^11^ using the MP and BI methods. Our analyses yielded a similar result; only the monophyly of Paederinae was supported, whereas the lineages of Staphylininae were recovered as polytomies. Based on this specific set of adult characters, the relationships between Paederinae and Staphylininae remained unresolved. The ‘paraphyly of Staphylininae’ proposed in Kypke *et al*.^11^ was not supported, however, because the sister relationship between Paederinae and (Platyprosopini, (Arrowinini, Staphylinini)) illustrated in Kypke *et al*.^11^ had no statistical support. The fact that the monophyly of Staphylininae was not recovered in our reanalysis nor in Kypke *et al*.^11^ does not necessarily indicate real polyphyly or monophyly of the subfamily, because the results were derived from a limited selection of adult characters, whereas monophyly of Staphylininae can be well-supported by the characters of their immature stages (obtect pupa, and larvae with large triangular eusternum on prothorax) as indicated by previous studies^1,3,7^.

Our new molecular phylogeny based on published DNA data provides strong evidence that both Paederinae and Staphylininae are monophyletic groups, as demonstrated in McKenna *et al*.^4^ and Grebennikov & Newton^3^. Compared with previously published results, our phylogenetic tree provides a more robust phylogeny of Staphylininae, shedding light on the early evolution of the subfamily.

### Intertribal relationships within staphylininae

We revealed a novel and robust phylogeny of Staphylininae, with the ML and BI methods generating the same topology: Platyprosopini (Arrowinini ((Diochini (Othiini (Maorothiini, Xantholinini))) Staphylinini)). All extant tribes were strongly supported as monophyletic, as in Solodovnikov & Newton^7^ and McKenna *et al*.^4^, but contradicting the results in Chatzimanolis *et al*.^12^ and Zhang & Zhou^16^.

The monogeneric tribe Platyprosopini was recovered as the earliest divergence of Staphylininae, which has been only found in a molecular phylogeny by Zhang & Zhou^16^, but not in any other molecular or morphology-based phylogenies. In contrast, Platyprosopini was supported as a sister group to the subfamily Paederinae in Brunke *et al*.^14^ and Schomann & Solodovnikov^17^, which made Schomann & Solodovnikov^17^ consider raising Platyprosopini to a subfamily. The incongruent results by the latter two studies likely resulted from the use of an insufficiently sequenced taxon (*Pseudopsis* sp.) as the outgroup. In morphology, extant species of *Platyprosopus* (Platyprosopini) are a peculiar group of Staphylininae: they have unique and probably plesiomorphic abdominal intersegmental membranes apparently with ‘brick-wall’ pattern (but actually of hexagonal sclerites). In addition, *Platyprosopus* have many other ancestral characters such as the absence of antesternal sclerites on prosternum, and trilobed aedeagus with a pair of long setose parameres^5^. Based on the combination of ancestral characters, Platyprosopini may be a ‘link’ between Staphylininae and Paederinae^31^.

Next to Platyprosopini, the monogeneric Arrowinini (*Arrowinus*) formed a sister group to the remaining tribes. Such a position of Arrowinini has never been found by any other phylogenetic analyses. Arrowinini was recovered as sister group to Staphylinini^7,11^, to Diochini^12^, to Platyprosopini^7^, to Platyprosopini + Paederinae^14,17^, or nested within Staphylinini^16^. Nevertheless, our results supported the long-standing hypothesis that *Arrowinus* is one of the most primitive and phylogenetically isolated lineages of Staphylininae^32^.

The Xantholinine-lineage^7,12^ minus Platyprosopini – (Diochini, Maorothiini, Othiini and Xantholinini) – formed a strongly supported monophyletic lineage in our trees. Although the interrelationships among these four tribes varied in different analyses, they were recovered together as monophyletic by morphological characters^6,7,11^ and some molecular data^16^, but not in other molecular phylogenies^4,12,14,17^. Monophyly of the four tribes is also well-supported by the presence of a large antesternal plate (or pair of large contiguous plates)^7,33^. As indicated in Solodovnikov & Newton^7^, members of Diochini have a larval synapomorphy: abdominal tergites II–VIII with anterior carinae becoming weaker on the more apical segments. In addition, the transparently sclerotized antesternal plates (equivalent to slightly sclerotized membrane in Newton *et al*.^5^) probably presents another potential synapomorphy of Diochini. The clade (Othiini, (Maorothiini, Xantholinini)) (MOX) recovered in our trees was strongly supported by other molecular phylogenies^4,14^. The monophyly of the tribes and their relationships were also confirmed in a larval character-based phylogeny^7^ and a phylogeny combining fossil and extant taxa^11^. It is noteworthy that Xantholinini was recovered as sister to Othiini (rather than Maorothiini) when the adult and larval characters were combined^7^. Consistent with the molecular phylogeny, monophyly of the tribes Othiini, Maorothiini and Xantholinini can be supported by the presence of one or two large sclerotized antesternal plate(s)^7^. Moreover, a sister relationship between Maorothiini and Xantholinini was supported by the triangular (not rectangular) antesternal plates^11^. The monophyly of each tribe of the Xantholinine lineage was well supported in all molecular and morphology-based phylogenies except Othiini in Kypke *et al*.^11^; this may result from insufficient sampling of morphological characters of the fossil species. Kypke *et al*.^11^ suggested that the morphology of *Vetatrecus* combined with the phylogenetic placement of Paederinae in their analyses questioned the placement of the MOX clade in Staphylininae, which may spur reconsideration of placing the clade in their own subfamily, a hypothesis first proposed by Thomson^34^. However, in our reanalyses using the same dataset and methods, the relationships among the MOX clade, other staphylinine tribes and Paederinae were not resolved at all, so it is misleading to overemphasize the importance of the discovery of an extinct genus for understanding the phylogeny of Staphylininae. Collectively, the striking incongruences between morphological and molecular data concerning the position of the ‘Xantholinini-lineage’ taxa indicated by the first molecular phylogeny of Staphylininae^12^ can be reconciled by our results based on sampling of more gene markers.

### Phylogeny of staphylinini

Monophyly of Staphylinini was strongly supported in our analyses, consistent with the results in Solodovnikov & Newton^7^ and Brunke *et al*.^14^. As noticed by Brunke *et al*.^14^, rejection of the monophyletic Staphylinini by Chatzimanolis *et al*.^12^ may have been an artefact of limited gene sampling, taxon sampling or both. The phylogenetic relationships within Staphylinini in our analyses were largely consistent with those in Brunke *et al*.^14^, but significantly different from Zhang & Zhou^16^, in which Staphylinini was recovered as paraphyletic. However, our trees were obviously better resolved than that in Brunke *et al*.^14^, especially for the deeper nodes. The earliest divergences within Staphylinini involved primarily south-temperate taxa, which was also supported by previous phylogenies^10,14^.

The South African endemic *Afroquedius* was weakly supported in ML tree as the first divergence within Staphylinini. By contrast, the relationship between *Afroquedius* and Amblyopinina supported in Brunke *et al*.^14^ was not supported by the morphology-based phylogeny of Brunke & Solodovnikov^10^, but was supported (with limited taxon sampling) in Solodovnikov and Schomann^9^. In our results, a sister group relationship of *Afroquedius* to Amblyopinina was not supported, although they share an aedeagus with a paramere closely affixed to the base of the median lobe^14^. Similarly, a basal position of the genus *Antimerus* (endemic to eastern Australia and Tasmania) was also supported by morphological characters^10^, but this differs from the hypothesis that it could be a basal member of ‘Staphylinini propria’^35^. It is notable that we cannot get a holistic picture of the early evolution of Staphylinini without other taxa of the ‘basal grade’^10^ such as the monotypic genus *Valdiviodes* Smetana from Chile, a genus unavailable for molecular study.

We recovered the subtribes Tanygnathinina and Amblyopinina as monophyletic, in congruence with other molecular phylogenies^12,14^. Unlike previous molecular phylogenies, a sister-group relationship between Tanygnathinina and Amblyopinina was recovered for the first time based on DNA data. Such a close affinity of the tribes was also found in many morphological phylogenies of Staphylinini, in which the Tanygnathinina (genus *Atanygnathus*) was deeply nested within Amblyopinina^9,10^. Interestingly, this relationship may also be supported by the discovery of the South African genus *Natalignathus* Solodovnikov (Amblyopinina), which was suggested to represent a missing phylogenetic link between Amblyopinina and Tanygnathinina^36^. Again, further gene sampling of transitional genera such as *Natalignathus* will be critical for better understanding the relationship between Tanygnathinina and Amblyopinina.

The relationships among the remaining lineages of Staphylinini (Cyrtoquediina, Erichsoniina, Acylophorina, Indoquediina, Quediina and Staphylinini propria) were identical to those found in Brunke *et al*.^14^, but slightly different from those in Chani-Posse *et al*.^15^, in which many deeper nodes were simply unsupported. Within Staphylinini propria, all subtribes, including Algonina, Anisolinina, Philothalpina, Philonthina, Staphylinina and Xanthopygina, were recovered as monophyletic, as found in Brunke *et al*.^14^. The recently established subtribe Cyrtoquediina comprises about 80 species, occurring in the Neotropical, Oriental or Palaearctic regions, except *Bolitogyrus* Chevrolat, which have a disjunct distribution in the first two regions^14,37,38^. The remarkable disjunct distribution of Cyrtoquediina suggests that this subtribe is probably ancient and relictual. Probably due to homoplasy, the monophyly of Quediina has not been well-resolved based on morphological characters^10^. The lineage ‘Staphylinini propria’ was recovered, congruent with previous phylogenetic reconstructions based on morphology^9,10^ and molecular data^12–14,16^.

### Molecular and morphology-based phylogenies: comparisons

When we re-analyzed the morphology-based datasets given in Solodovnikov & Newton^7^ and Kypke *et al*.^11^ using the MP and BI approaches, our results were largely consistent with the original results. However, we found that the trees in Solodovnikov & Newton^7^ and Kypke *et al*.^11^ were shown as generally much better resolved than our trees, but this is because clades that were statistically unsupported were not collapsed in their trees. The unsupported topologies thus result in a misleading interpretation of the evolution of Staphylininae. For example, Paederinae was shown as a sister group to Platyprosopini + Arrowinini + Staphylinini in Kypke *et al*.^11^, but this relationship was not actually supported in either our results or those of Kypke *et al*.^11^. Fossils are important for understanding early evolution of Staphylininae, but overemphasis of their significance and disregarding the robustness (support values) of phylogenies, as in Solodovnikov *et al*.^2^ and Kypke *et al*.^11^, may generate incorrect conclusions.

We also found that the morphology-based phylogenies were generally worse resolved than the DNA-based phylogeny. Based on the existing morphology-based datasets of Staphylininae, with or without fossil taxa, the intertribal relationships within Staphylininae cannot be completely resolved. The molecular phylogenies based on multiple genes as here analyzed are of great significance for understanding the character evolution, biogeography, and ecology of the diverse subfamily Staphylininae. In addition, without a robust phylogeny of Staphylininae, timetrees of the subfamily such as shown in Brunke *et al*.^39^ cannot be reconstructed with confidence.

## Conclusions

We present a robust phylogeny of the rove beetle subfamily Staphylininae based on extensive sampling of genes and taxa from GenBank. The well-resolved molecular phylogeny of Staphylininae agreed with the majority of previous morphology-based studies, except Kypke *et al*.^11^, in which the deeper nodes were demonstrated as unsupported by the present study. Monophyly of Staphylininae as conventionally defined was strongly supported, rejecting the hypothesis of paraphyly recently proposed by Brunke *et al*.^14^, Schomann & Solodovnikov^17^ and Kypke *et al*.^11^. A new pattern of intertribal relationships within Staphylininae: Platyprosopini (Arrowinini ((Diochini (Othiini (Maorothiini, Xantholinini))) Staphylinini)) was strongly supported. Further sampling of more genes and taxa of Staphylininae (especially key taxa from the southern hemisphere) will undoubtedly provide further insights into the phylogeny and early evolution of the subfamily.

## Supplementary information

Supplementary
