# Supplementary material for: Congruence of morphological and molecular phylogenies of the rove beetle subfamily Staphylininae (Coleoptera: Staphylinidae)
Source: Sci Rep. 2019 Oct 22;9:15137. doi: 10.1038/s41598-019-51408-1 (PMC6805933; doi:10.1038/s41598-019-51408-1)
Supplement: Supplementary file 1 — Supplementary [file 41598_2019_51408_MOESM1_ESM.pdf]

Congruence of morphological and molecular phylogenies of the rove beetle subfamily Staphylininae (Coleoptera: Staphylinidae)

CHEN-YANG CAI[1,2], YONG-LI WANG[3], LIANG LÜ[4], ZI-WEI YIN[5], MARGARET K. THAYER[6], ALFRED F. NEWTON[6] and YU-LINGZI ZHOU[7,8]

[1]State Key Laboratory of Palaeobiology and Stratigraphy, Nanjing Institute of Geology and Palaeontology and Center for Excellence in Life and Paleoenvironment, Chinese Academy of Sciences, Nanjing 210008, China, [2]School of Earth Sciences, University of Bristol, Life Sciences Building, Tyndall Avenue, Bristol BS8 1TQ, UK, [3]Biofuels Institute, School of the Environment and Safety Engineering, Jiangsu University, Zhenjiang 212013, China, [4]College of Life Sciences, Hehei Normal University, Shijiazhuang 050024, China, [5]Lab of Environmental Entomology, College of Life Sciences, Shanghai Normal University, Shanghai 200234, China, [6]Integrative Research Center, Field Museum of Natural History, Chicago, IL 60605, USA, [7]Key Laboratory of Zoological Systematics and Evolution, Institute of Zoology, Chinese Academy of Sciences, Beijing, China, and [8]Australian National Insect Collection, CSIRO, Canberra, Australia

Correspondence: Chen-Yang Cai, Nanjing Institute of Geology and Palaeontology, Chinese Academy of Sciences, No.39 East Beijing Road, Nanjing 210008, China. E-mail: cycai@nigpas.ac.cn; Yu-lingzi Zhou, Australian National Insect Collection, CSIRO, GPO Box 1700, Canberra, ACT 2601, Australia. E-mail: Lingzi.Zhou@csiro.au

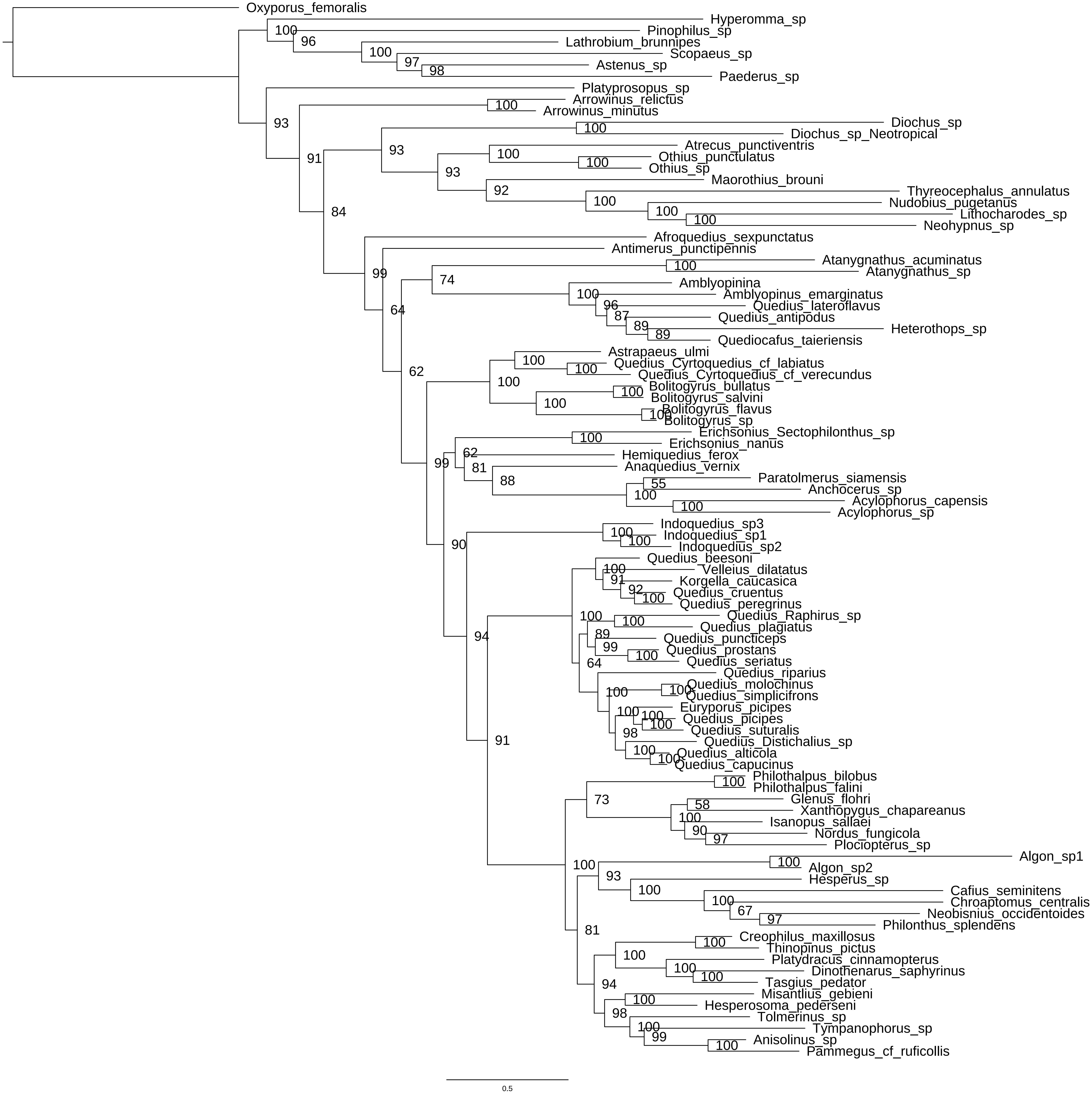
Oxyporus_femoralis
Hyperomma_sp
Pinophilus_sp
Lathrobium_brunnipes
Scopaeus_sp
Astenus_sp
Paederus_sp
Platyprosopus_sp
Arrowinus_relictus
Arrowinus_minutus
Diochus_sp
Diochus_sp_Neotropical
Atrecus_puncticentris
Othius_punctulatus
Othius_sp
Maorothius_brouni
Thyreocephalus_annulatus
Nudobius_pugetanus
Lithocharodes_sp
Neohypnus_sp
Afroquedius_sexpunctatus
Antimerus_punctipennis
Atanygnathus_acuminatus
Atanygnathus_sp
Amblyopinina
Amblyopinus_emarginatus
Quedius_lateroflavus
Quedius_antipodus
Heterothops_sp
Quediocafus_taieriensis
Astrapaeus_ulmi
Quedius_Cyrtoquedius_cf_labiatus
Quedius_Cyrtoquedius_cf_verecundus
Bolitogyrus_bullatus
Bolitogyrus_salvini
Bolitogyrus_flavus
Bolitogyrus_sp
Erichsonius_Sectophilonthus_sp
Erichsonius_nanus
Hemiquedius_ferox
Anaquedius_vernix
Paratolmerus_siamensis
Anchocerus_sp
Acylophorus_capensis
Acylophorus_sp
Indoquedius_sp3
Indoquedius_sp1
Indoquedius_sp2
Quedius_beesoni
Velleius_dilatatus
Korgella_caucasica
Quedius_cruentus
Quedius_peregrinus
Quedius_Raphirus_sp
Quedius_plagiatus
Quedius_puncticeps
Quedius_prostans
Quedius_seriatus
Quedius_riparius
Quedius_molochinus
Quedius_simplicifrons
Euryporus_picipes
Quedius_picipes
Quedius_suturalis
Quedius_Distichalius_sp
Quedius_alticola
Quedius_capucinus
Philothalpus_bilobus
Philothalpus_falini
Glenus_flohri
Xanthopygus_chapareanus
Isanopus_sallaei
Nordus_fungicola
Plociopterus_sp
Algon_sp1
Algon_sp2
Hesperus_sp
Cafius_seminitens
Chroaptomus_centralis
Neobisnius_occidentoides
Philonthus_splendens
Creophilus_maxillosus
Thinopinus_pictus
Platydracus_cinnamopterus
Dinothenarus_saphyrinus
Tasgius_pedator
Misantlius_gebieni
Hesperosoma_pederseni
Tolmerinus_sp
Tympanophorus_sp
Anisolinus_sp
Pammegus_cf_ruficollis
0.5

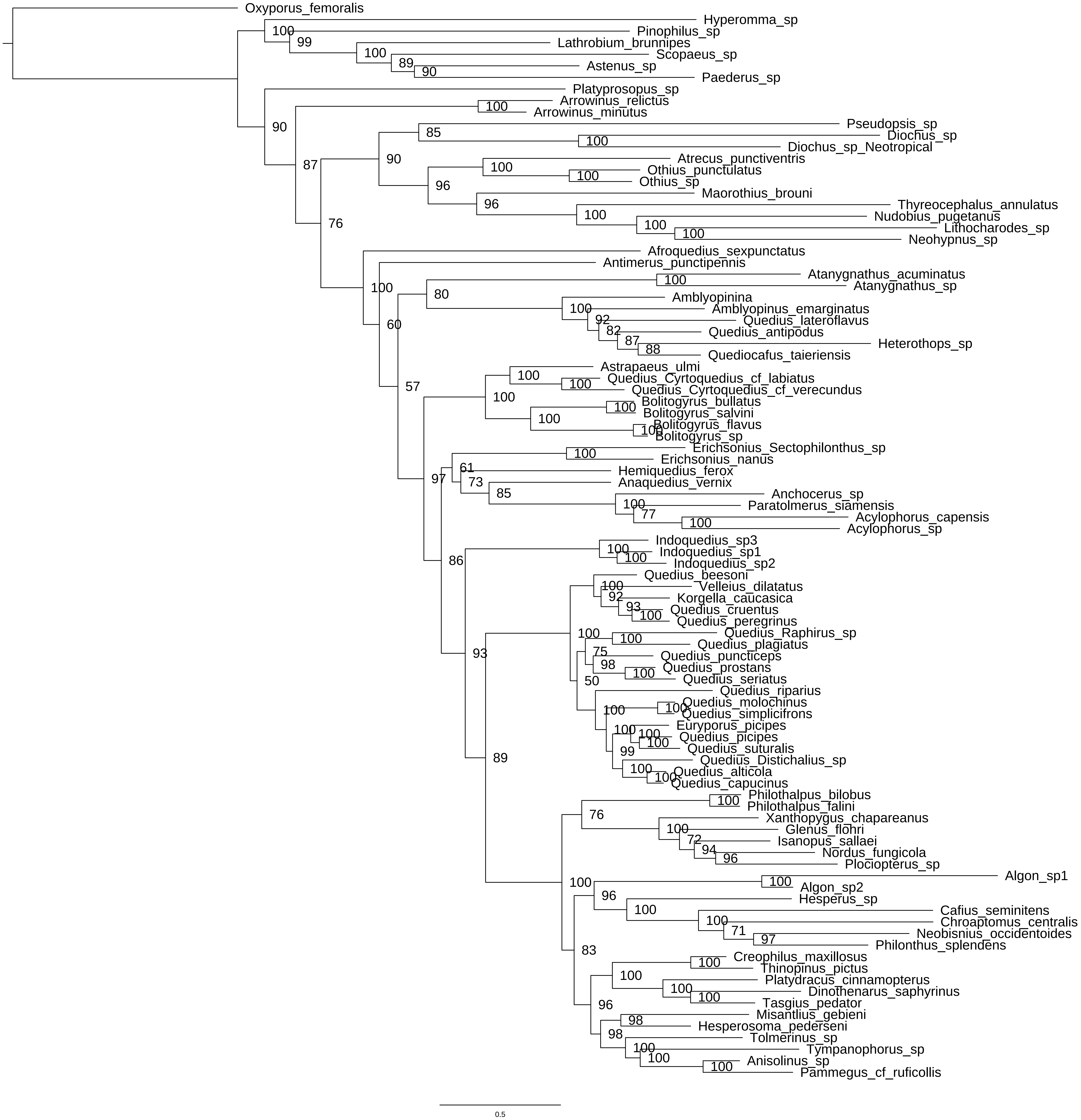
Oxyporus_femoralis
Hyperomma_sp
Pinophilus_sp
Lathrobium_brunnipes
Scopaeus_sp
Astenus_sp
Paederus_sp
Platyprosopus_sp
Arrowinus_relictus
Arrowinus_minutus
Pseudopsis_sp
Diochus_sp
Diochus_sp_Neotropical
Atrecus_punctiventris
Othius_punctulatus
Othius_sp
Maorothius_brouni
Thyreocephalus_annulatus
Nudobius_pugetanus
Lithocharodes_sp
Neohypnus_sp
Afroquedius_sexpunctatus
Antimerus_punctipennis
Atanygnathus_acuminatus
Atanygnathus_sp
Amblyopinina
Amblyopinus_emarginatus
Quedius_lateroflavus
Quedius_antipodus
Heterothops_sp
Quediocafus_taieriensis
Astrapaeus_ulmi
Quedius_Cyrtoquedius_cf_labiatus
Quedius_Cyrtoquedius_cf_verecundus
Bolitogyrus_bullatus
Bolitogyrus_salvini
Bolitogyrus_flavus
Bolitogyrus_sp
Erichsonius_Sectophilonthus_sp
Erichsonius_nanus
Hemiquedius_ferox
Anaquedius_vernix
Anchocerus_sp
Paratolmerus_siamensis
Acylophorus_capensis
Acylophorus_sp
Indoquedius_sp3
Indoquedius_sp1
Indoquedius_sp2
Quedius_beesoni
Velleius_dilatatus
Korgella_caucasica
Quedius_cruentus
Quedius_peregrinus
Quedius_Raphirus_sp
Quedius_plagiatus
Quedius_puncticeps
Quedius_prostans
Quedius_seriatus
Quedius_riparius
Quedius_molochinus
Quedius_simplicifrons
Euryporus_picipes
Quedius_picipes
Quedius_suturalis
Quedius_Distichalius_sp
Quedius_alticola
Quedius_capucinus
Philothalpus_bilobus
Philothalpus_falini
Xanthopygus_chapareanus
Glenus_flohri
Isanopus_sallaei
Nordus_fungicola
Plociopterus_sp
Algon_sp1
Algon_sp2
Hesperus_sp
Cafius_seminitens
Chroaptomus_centralis
Neobisnius_occidentoides
Philonthus_splendens
Creophilus_maxillosus
Thinopinus_pictus
Platydracus_cinnamopterus
Dinothenarus_saphyrinus
Tasgius_pedator
Misantlius_gebieni
Hesperosoma_pederseni
Tolmerinus_sp
Tympanophorus_sp
Anisolinus_sp
Pammegus_cf_ruficollis
0.5
